# Elucidating the Beneficial Role of PPAR Agonists in Cardiac Diseases

**DOI:** 10.3390/ijms19113464

**Published:** 2018-11-04

**Authors:** Zaza Khuchua, Aleksandr I. Glukhov, Arnold W. Strauss, Sabzali Javadov

**Affiliations:** 1 The Heart Institute, Cincinnati Children’s Hospital Medical Center, Cincinnati, OH, 45229-7020,USA; arnold.strauss@cchmc.org; 2 Department of Biochemistry, Sechenov University, Moscow, 119991 Russia; aiglukhov1958@gmail.com; 3 Department of Biochemistry, Ilia University, Tbilisi, 0162 Georgia; 4 Department of Biology, Lomonosov Moscow State University 119991, Moscow, Russia; 5 Department of Physiology, University of Puerto Rico School of Medicine, San Juan, PR, 00936-5067, USA

**Keywords:** PPAR agonists, bezafibrate, heart, cardiomyopathy, heart failure, lipids, fatty acid oxidation, energy metabolism, mitochondria

## Abstract

Peroxisome proliferator-activated receptors (PPARs) are nuclear hormone receptors that bind to DNA and regulate transcription of genes involved in lipid and glucose metabolism. A growing number of studies provide strong evidence that PPARs are the promising pharmacological targets for therapeutic intervention in various diseases including cardiovascular disorders caused by compromised energy metabolism. PPAR agonists have been widely used for decades as lipid-lowering and anti-inflammatory drugs. Existing studies are mainly focused on the anti-atherosclerotic effects of PPAR agonists; however, their role in the maintenance of cellular bioenergetics remains unclear. Recent studies on animal models and patients suggest that PPAR agonists can normalize lipid metabolism by stimulating fatty acid oxidation. These studies indicate the importance of elucidation of PPAR agonists as potential pharmacological agents for protection of the heart from energy deprivation. Here, we summarize and provide a comprehensive analysis of previous studies on the role of PPARs in the heart under normal and pathological conditions. In addition, the review discusses the PPARs as a therapeutic target and the beneficial effects of PPAR agonists, particularly bezafibrate, to attenuate cardiomyopathy and heart failure in patients and animal models.

## 1. Introduction

**Figure 1:**
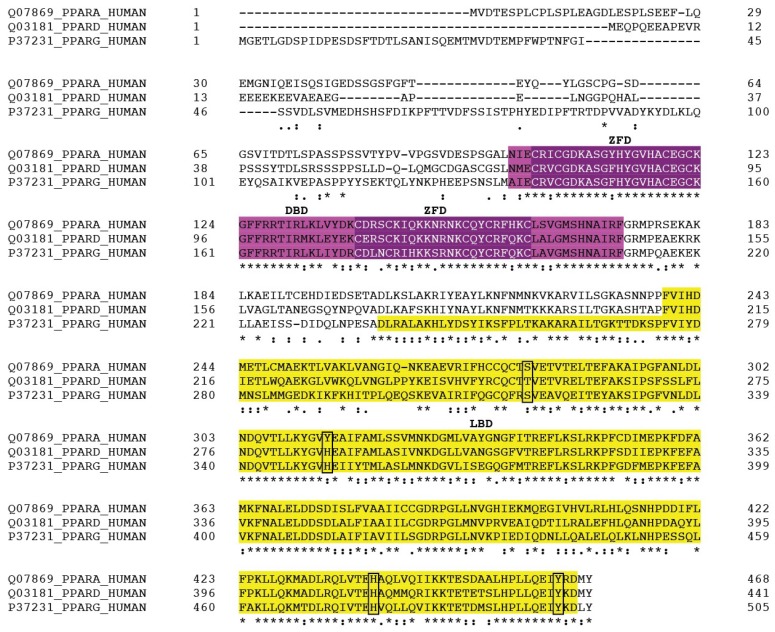
Amino acid sequence alignments of human peroxisome proliferator-activated receptors (PPAR) isoforms. DNA-binding domain (DBD, purple), zinc-finger domains (ZFD, purple), and ligand binding domain (LBD, yellow) are highlighted. All three isoforms of PPAR possess a high degree of inter-species sequence homology, particularly in the DBD and LBD. The sequence positions that are conserved within PPAR isoforms are important for identification of the structural dynamics, ligand affinity, and DNA binding specificity. Amino acid residues, which participate in ligand binding, are boxed. Alignment was performed with CLUSTALO (https://www.uniprot.org/align/). (*)—fully conserved residues; (:)—residues with strongly similar properties; (.)—residues with weakly similar properties.

Peroxisome proliferator-activated receptors (PPARs) play an important role in the regulation of carbohydrate and lipid metabolism in the cell. They are involved in the transcriptional regulation of multiple processes and play a central role in the pathogenesis of metabolic disorders, cardiovascular diseases, diabetes, cancer, inflammation, and other diseases. PPARs are members of the nuclear hormone receptor superfamily and act as ligand-activated transcription factors. They were first discovered in the early 1990s as transcription factors that mediate proliferation of peroxisomes in the cell [1,2,3]. Interestingly, biological effects of bezafibrate (BF), a potent pan-specific activator of PPARs, were demonstrated before cloning and discovery of PPARs [4,5,6]. Currently, there are three PPAR isoforms, PPARα, PPARβ/δ, and PPARγ that are encoded by separate genes. All three isoforms possess a high degree of inter-species sequence homology, particularly in the DNA-binding domain (DBD) and ligand-binding domain (LBD) [7,8] (Figure 1). The central role of PPARs in heart metabolism, particularly fatty acid oxidation (FAO) and mitochondrial bioenergetics, makes them a promising therapeutic target for the treatment of cardiac diseases, such as myocardial infarction (MI) and heart failure (HF). A growing number of studies using experimental animal models and patients often provide controversial data on the beneficial effects of PPAR agonists in cardiac diseases. In this review, we summarize and discuss the role of PPARs, particularly PPARα, in the healthy heart and cardiac diseases. In addition, we provide a comprehensive discussion of PPAR agonists in the treatment of cardiac diseases, particularly cardiomyopathy and HF. 

## 2. Biological Role and Tissue-Specific Expression of PPARs

**Figure 2:**
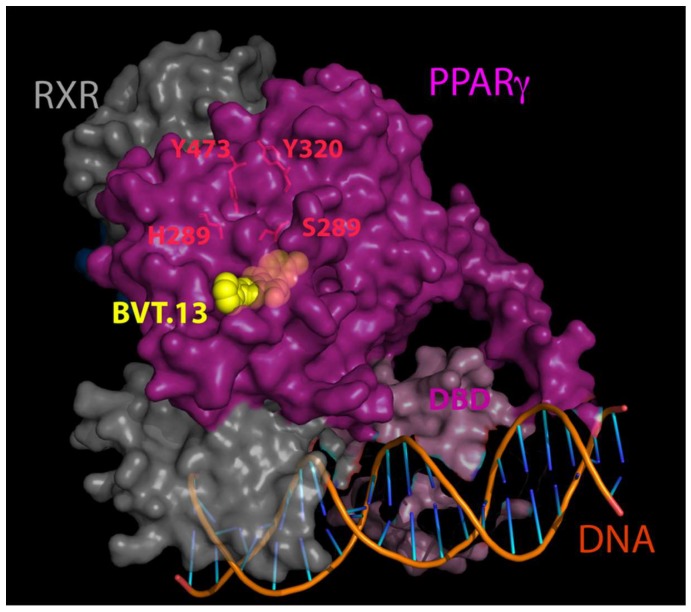
X-ray crystal structure of the complex of PPARγ (magenta) and retinoid X receptors (RXR) (grey) at 3.2 Å resolution. The BVT.13 agonist ligand is displayed as yellow balls. The amino acids residues, which form a ligand binding pocket, are shown in *red*. DNA-binding domain (DBD, light magenta) and DNA fragment are shown. The structure is derived from Protein Data Bank (PDB: 3DZU) [12] and visualized using PyMol software (v. 2.0.7).

PPARs form heterodimers with the retinoid X receptor in the nucleus. These heterodimers recruit coactivators and corepressors and bind to specific peroxisome proliferator response elements (PPRE) in regulatory regions of PPAR target genes (Figure 2). Ligand binding releases the corepressor from the complex and allows activation of coactivator leading to changes in target gene expression [9]. DNA-pull down of PPARγ with subsequent MS-based proteomics identification of binding partners revealed highly complex patterns of interaction of PPARγ with other proteins in the cytoplasm and nucleus [10]. In addition, this study revealed that interactions of PPARγ with its binding partners are highly ligand- and DNA-dependent. In silico analysis of protein–protein interactions between PPARα and PPARβ/δ predicted the interaction of PPARs and retinoid X receptors (RXRs) with chromatin state modifiers, such as histone deacetylases (HDACs) that can play a role in epigenetic modifications of the diseases [11].

PPARα plays a crucial role in the regulation of FAO, a major source of ATP in high energy-consuming organs and tissues. Hence, PPARα is highly expressed in skeletal muscle, heart, liver, and brown adipose tissue [13,14,15]. PPARγ is mainly expressed in adipose tissue, large intestine, and spleen. It regulates adipogenesis, lipid and glucose metabolism, and inflammatory pathways. The least studied PPARβ/δ is expressed ubiquitously with the highest levels found in the liver, intestine, kidney, adipose tissue, and skeletal muscle thereby, suggesting its fundamental role in cellular biology (reviewed in References [16,17,18,19]).

Transcriptional activities of PPARs are regulated, in part, by the PPARγ coactivator 1α (PGC-1α). PGC-1α is an integrator of the transcriptional network regulating mitochondrial biogenesis and, like PPARα, highly expressed in high energy-consuming cells. In addition to PPARs, PGC-1α mediates its effects through other downstream transcriptional regulatory circuits such as estrogen-related receptors (ERRs), and nuclear respiratory factors (NRF) 1 and 2. The nuclear respiratory factors, in turn, regulate downstream genes, including mitochondrial transcription factor A (TFAM), which is responsible for the maintenance as well as replication and transcription of mitochondrial DNA (reviewed in References [20,21,22]). Thus, PGC-1α is an inducible co-activator that coordinately regulates mitochondrial biogenesis through the network of transcription factors PPARs/NRF/ERRs. Mitochondrial biogenesis via the PGC-1α/NRF pathway is apparently regulated by AMP kinase (AMPK) [23,24]. Indeed, direct phosphorylation of PGC-1α by AMPK in vitro and in cultured cells has been shown recently [25].

Various natural fatty acids and eicosanoids act as natural ligands for PPARs. Generally, polyunsaturated fatty acids (PUFAs) display a higher affinity to PPARγ and PPARβ/δ, while both saturated and unsaturated fatty acids interact with PPARα equally efficiently (reviewed in Reference [8]). Therefore, PPARs represent attractive molecular targets for the development of pharmacological agents and treatment of metabolic disorders, including obesity, type 2 diabetes, dyslipidemia, and cardiovascular diseases.

## 3. The Role of PPARs in Cardiac Diseases

PPARα is highly expressed in cardiomyocytes, and genetic studies demonstrated the importance of PPARα in fatty acid metabolism in the heart [26,27]. PPARα knockout mice demonstrated normal [27] or reduced [26,28] cardiac function. Cardiac dysfunction in PPARα^−/−^ mice was associated with structural abnormalities in mitochondria [26] and increased oxidative stress due to downregulation of the antioxidant capacity in the heart [29]. High workload decreased cardiac performance in PPARα knockout mice associated with lower levels of ATP in the myocardium [30]. In response to transverse aortic constriction (TAC), PPARα-null mice showed pronounced cardiac hypertrophy [31]. On the other hand, overexpression of PPARα increased mild cardiac hypertrophy, ventricular dysfunction, and lipotoxicity associated with reciprocal repression of glucose uptake and oxidation in the mouse heart [32]. These mice developed a phenotype strikingly similar to diabetic cardiomyopathy [33]. The contrasting metabolic phenotypes induced by genetic upregulation or downregulation of PPARα in mice indicate the central role of the receptors in regulating glucose and lipid metabolism in the heart.

Recent studies demonstrated that the expression of PPARα significantly decreases in cardiomyocytes in a pressure–overload mouse model of HF induced by TAC. Expression of PPARα target genes, carnitine palmitoyltransferase 1 (CPT-1) and fatty acid transport protein 1 (FATP1) were also significantly reduced in the HF hearts. Activation of PPARα either by cardiac-specific overexpression of *PPARα* gene or by treating mice with the specific PPARα agonist, WY-1463 improved cardiac function, attenuated cardiac fibrosis, and preserved FAO and high-energy phosphates in a mouse model of HF induced by TAC [34]. The energy substrate switch from FAO to glucose oxidation and other metabolic changes in hearts with hypertrophy and HF is mediated, at least in part, through downregulation of genes encoding FAO and oxidative phosphorylation enzymes due to deactivation of the PGC-1α/PPARα pathway [21,22]. Reduced PGC-1α and PPARα expression occurs in animal models of HF [35,36] and in failing human hearts [37,38], suggesting that deactivation of the PGC-1α/PPARα pathway in the failing heart plays a critical role in coincident mitochondrial dysfunction. 

The role of post-translational modifications (PTMs) in activation/inactivation of PPARα is debated. PPARs have been shown to undergo several types of PTMs including phosphorylation, acetylation, sumoylation, and ubiquitination, among others. Several protein kinases, including extracellular signal-regulated protein kinases 1 and 2 and c-Jun N-terminal kinase, AMPK, protein kinase A, and glycogen synthase kinase 3 phosphorylate PPARα and PPARγ [39]. Protein kinase A [40] and p38 [41] phosphorylated PPARα that resulted in a ligand-dependent increase of PPARα activity in neonatal rat cardiomyocytes and HEK-293 cells. On the other hand, ventricular pressure overload in mice and PPARα overexpression in cardiomyocytes revealed that downregulation of cardiac PPARα and alteration of its activity during hypertrophic growth occur at the posttranscriptional level via activation of extracellular signal-regulated protein kinases 1 and 2 [42]. Phosphorylation increased transcriptional activation of PPARα [43] but decreased that of PPARγ [44].

PPARα seems to be a downstream target for AMPK and mediates its beneficial effects by improving mitochondrial metabolism. AMPK is the main cellular energy sensor that initiates ATP generating processes while blocking ATP consuming processes. It is also involved in the regulation of mitochondrial metabolism and the redox state in the cell (reviewed in References [45,46]). Pharmacological activation of AMPK stimulates FAO through increased expression of PPARα target genes in skeletal muscle cells [47]. Furthermore, the PPARα inhibitor, GW6471, prevented the cardioprotective effects of metformin, an AMPK activator, against ischemia-reperfusion in rat hearts [48]. Oxidative stress-induced phosphorylation of PGC-1α and PPARα in cardiac cells. However, the protective effects of the AMPK activators metformin and A-769662 on hydrogen peroxide-treated H9c2 cells and in vivo cardiac ischemia-reperfusion in rats were not associated with phosphorylation of PPARα [49,50]. These studies suggest that PTMs of PPARα during cardiac oxidative stress and hypertrophic growth can occur at several levels. 

In addition to regulation of the mitochondrial transcriptional network, PPARα can translocate to mitochondria and affect metabolism and function of mitochondria. Hydrogen peroxide-induced oxidative stress in H9c2 cells [49] and ischemia-reperfusion in the rat heart [50] stimulated protein-protein interactions between PPARα and cyclophilin D (CyP-D), a major regulator of the mitochondrial permeability transition pore. The interaction provoked the opening of the mitochondrial permeability transition pores. Conversely, activation of AMPK with metformin or A-769662 prevented PPARα-CyP-D interaction leading to inhibition of mitochondrial permeability transition pore opening, and improved cell survival and post-infarction recovery [49,50]. These studies indicate the role of PPARα in mediating the beneficial effects of AMPK in cardiac ischemia-reperfusion. 

Similar to PPARα, heart-specific PPARγ knockout mice developed cardiac hypertrophy with preserved normal cardiac metabolism and function [51,52]. Decreased expression of genes encoding FAO enzymes and impaired fatty acid utilization with unchanged glucose oxidation were found in inducible cardiomyocyte-specific PPARγ^−/−^ mice [53]. It should be noted that cardiac hypertrophy in heart-specific PPARγ knockout mice associated with oxidative damage and mitochondrial dysfunction progresses with age and leads to dilated cardiomyopathy and premature death [54]. Like PPARα^−/−^ mice, antioxidant therapy attenuated cardiac dysfunction in the PPARγ^−/−^ mice. Heart-specific PPARγ overexpression induced a dilated cardiomyopathy associated with increased expression of FAO genes, lipotoxicity, and mitochondrial structural abnormalities such as cristae disruption in the heart [55]. Interestingly, glucose uptake was not decreased in these hearts. 

Overexpression of PPARβ/δ in mouse hearts enhanced mitochondrial biogenesis, myocardial oxidative metabolism, improved cardiac performance, and reduced cardiac fibrosis [56]. These effects of PPARβ/δ overexpression were not affected by TAC-induced cardiac hypertrophy. In rats with congestive HF, the PPARβ/δ-specific agonist, GW610742X, normalized cardiac substrate metabolism in a dose-dependent manner, dramatically reduced right ventricular hypertrophy, and decreased the level of the arterial natriuretic peptide in the right ventricle. However, GW610742X had no beneficial effect on the left ventricular function [57,58].

The activity of a large number of proteins is regulated through acetylation/deacetylation of lysine residues. Four classes of HDACs play a central role in cell metabolism, including energy metabolism in the heart. Mitochondrial bioenergetics including fatty acid metabolism, electron transfer chain, and oxidative phosphorylation are regulated by the class III HDACs sirtuins, particularly SIRT3 [59]. Interestingly, the interaction of HDAC3 with PPARγ induced deacetylation of the protein and reduced its activity [60]. Inhibition of HDAC3 stimulated ligand-independent activation of PPARγ by protein acetylation suggesting that acetylation of PPARγ induces its activation through a ligand-independent mechanism. Cardiac-specific HDAC3 knockout mice demonstrated a modest increase in expression of FAO genes with no changes in gene expression of PPARs [61]. Oxidative stress induced by hydrogen peroxide did not increase acetylation of PGC-1α and PPARα in H9c2 cardioblasts [49]. Further studies are needed to establish a cause–effect relationship between acetylation and activity of PPARs in the healthy heart and cardiac diseases. 

Other forms of PTM, such as sumoylation [62] and ubiquitination [63], have been shown to affect the PPAR activity (reviewed in Reference [58]). These studies were conducted mostly using various cell lines, and there are few, if any, studies on the PPAR sumoylation and ubiquitination in the heart. For instance, upregulation of the ubiquitin ligase, muscle ring finger-1 increased its interaction and ubiquitination of PPARα in neonatal cardiomyocytes [64]. The ubiquitination reduced PPARα activity and FAO suggesting a critical role of ubiquitination in regulating cardiac PPARα and fatty acid metabolism in the heart.

Polymorphisms in PPARs are significantly associated with cardiac disorders. Intronic rs4253778 polymorphism and common L162V (rs1800206) polymorphism in *PPARα* are significantly associated with coronary heart disease (CHD) risk [65]. L162V variant at the DBD region of PPARα affects the transactivation activity of PPAR ligands [66,67]. A Rs135551 intronic variant in *PPARα* showed significant association with CHD [68]. T allele carriers of C161T polymorphism in *PPARγ* (rs3856806) have lower CHD risk, but higher risk of acute coronary syndrome (ACS). +294T/C polymorphism at *PPARγ*/*δ* (rs2016520) is significantly associated with ACS [65]. 

Thus, PPARs play an important role in fatty acid metabolism in the heart and are involved in the pathogenesis of cardiac hypertrophy, cardiomyopathy, and HF. Apparently, beneficial or detrimental effects of PTMs of PPARα depend on the severity and timing of oxidative and energy stresses that are associated with the diminished capacity of the myocardium to maintain lipid and glucose metabolism.

## 4. Therapeutic Potential of PPAR Agonists in Cardiac Diseases

### 4.1. Studies in Animal Models

Lower rates of FAO are associated with cardiomyopathies and HF [69,70,71,72,73]. Due to high expression and the beneficial role of PPARα and PPARβ/δ in the heart, numerous studies have been conducted to study the efficiency of PPARα and PPARβ/δ agonists on various animal models with HF. 

**Figure 3:**
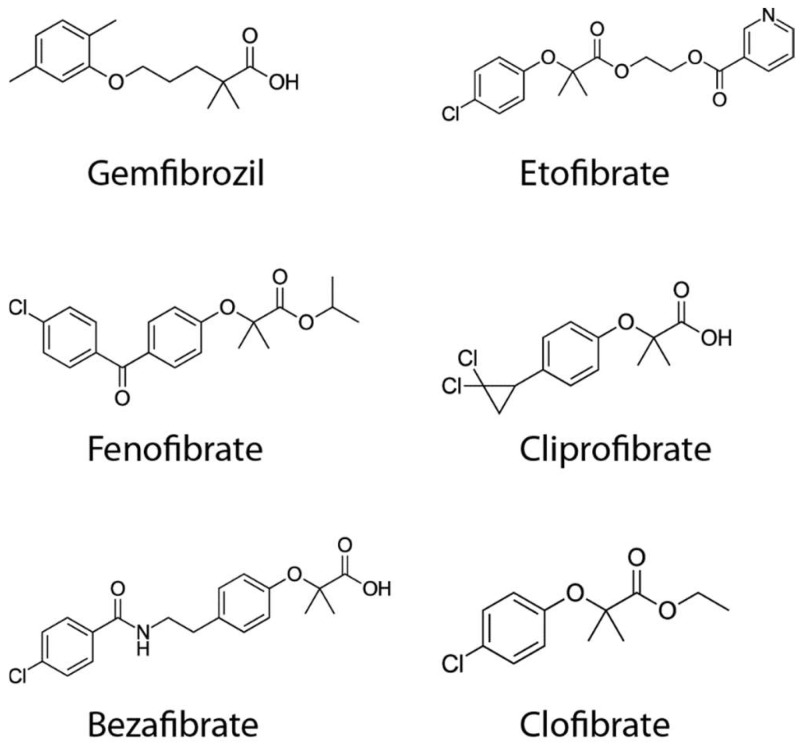
Chemical structures of fibrates.

Fibrates have been used for many years as PPAR agonists for treatment heart attacks and strokes. The fibrates are a family of hypolipidemic drugs that are structural derivatives of the parent compound, clofibrate (ethyl 2-(4-chlorophenoxy)-2-methylpropionate (Figure 3). They lower serum triglycerides, raise high-density lipoprotein cholesterol (HDL-C) and lower low-density lipoprotein cholesterol (LDL-C) levels. Therefore, long-term therapy with fibrates could help to prevent cardiovascular disease events. However, fibrates demonstrate a high risk for developing rhabdomyolysis and renal failure (reviewed in Reference [74]). The main list of fibrates currently used in experimental studies and clinical trials include gemfibrozil, fenofibrate, BF, etofibrate, and ciprofibrate (Figure 3). The clofibrate previously used in studies is no longer in use due to safety concerns. 

Fenofibrate is a dual activator of PPARα and PPARγ, with 10-fold selectivity for PPARα [18]. Oral intake of fenofibrate (100 mg/kg body weight) significantly attenuated end-diastolic and end-systolic left ventricular dimensions and cardiac fibrosis in aldosterone-induced hypertrophy model independently of an effect of the drug on blood pressure [75]. Similar effects were observed in porcine and canine tachycardia-induced cardiomyopathy models [76,77]. Fenofibrate attenuated hypertrophy, inhibited the inflammatory response, improved the survival of Dahl salt-sensitive rats [78] and decreased fibrosis in the rat model with the pressure overload-induced HF [79]. While fenofibrate had clear beneficial effects in wild-type mice, this drug had deleterious consequences on cardiac hypertrophy and fibrosis in PPARα^−/−^ mice [80]. The detrimental effects of fenofibrate in PPARα knockout mice might be a result of anomalous activation of PPARγ and PPARβ/δ in cardiomyocytes in the absence of PPARα. Administration of another PPARα agonist, tetradecylthioacetic acid, elevated expression of PPARα target genes, myocardial oxygen consumption, and FAO with concomitant reduction of glucose oxidation in the heart. However, this drug had a negative impact on the post-ischemic recovery of cardiac function in an isolated perfused heart model [81].

Fibrates require micromolar concentrations to activate PPARα. The half maximal effective concentration (EC_50_) for fenofibrate is approximately 30 μM for human PPARα [18] that requires high doses of the drug (>100 mg/kg) to achieve a clinical effect. Attempts to discover more potent and more selective PPARα agonists resulted in the synthesis of several more potent compounds that work in nanomolar ranges. The PPARα agonist AVE8134 has a high affinity for PPARα (EC_50_ 0.01 and 0.03 μM for human and rodent PPARα, respectively). AVE8134 at the daily dose of 3–30 mg/kg improved lipid profile and augmented glucose metabolism; prevented post-MI hypertrophy, fibrosis and cardiac dysfunction, and reduced mortality in rats [82,83]. Other selective PPARα agonists, WY-14643 (pirinixic acid) and GW7647, have EC_50_ in the micromolar range. WY-14643 (0.01% *w*/*w* in rodent food, or ~20 mg/kg) significantly attenuated cardiac dysfunction and remodeling induced by pressure–overload HF in mice [84]. 

Treatment with GW7647 did not prevent the development of hypertrophy but preserved the left ventricular ejection fraction during pressure–overload cardiac hypertrophy in rabbits [85]. The effects of GW7647 were associated with an increased cardiac FAO and overall ATP production that resulted in an improved post-ischemic recovery of cardiac function. In addition, GW7647 treatment resulted in relived endoplasmic reticulum stress, preserved mitochondrial membrane potential, and activated sarcoplasmic reticulum Ca-ATPase (ATP2A2) [85]. Additionally, GW2331 and GW9578, dual PPARα/PPARγ agonists that work in the nanomolar range, have been synthesized [86,87].

Bezafibrate was introduced as a lipid-lowering drug by Boehringer Mannheim in 1970s [6,88]. It reduces heart rate, blood pressure, insulin level, and free fatty acids in patients with hypertriglyceridemia [89]. Bezafibrate also is a widely used pan-PPAR agonist in animal trials. It activates all three PPAR subtypes with the highest affinity for PPARα and PPARβ/δ isoforms [90]. The EC_50_s for BF are 50, 60, and 20 μM for human PPARα, PPARγ, and PPARβ/δ, respectively; and 90, 55, and 110 μM for mouse PPARα, PPARγ, and PPARβ/δ, respectively [18]. There are significant inconsistencies in BF studies in humans and rodents. In clinical practice, BF is typically prescribed at a daily dose of 10–25 mg/kg [6,91,92]. Conversely, in animal studies BF is usually administrated *per os* with diet in the amount of 0.5% w/w, corresponding to a daily dose of 600–800 mg per kg [93,94,95]. It is conceivable that the relatively low affinity of BF for murine PPARβ/δ requires a higher dose of the drug to achieve a biological response in mice, particularly in skeletal muscle, where the BF effects are predominantly mediated by PPARβ/δ rather than PPARα [96].

Experiments with PPARα knockout mice suggested that more clinically relevant, low-dose BF decreases serum and liver triglycerides in a PPAR-independent manner by suppressing the expression of sterol regulatory element-binding protein 1c (*SPREBP1*) affecting hepatic lipogenesis and triglyceride secretion [97]. Several groups have reported on the use of BF in mouse models with varying success. Two mouse models of cytochrome *c*-oxidase deficiency, systemic *Surf1^−/−^* and muscle-specific *Cox15^−/−^*, were given BF at the dose of 0.5% in rodent diet (Table 1). At this dose, BF was highly toxic, causing massive apoptosis in skeletal muscles. In these models, BF induced expression of the genes encoding proteins that are involved in FAO but not oxidative phosphorylation in mitochondria. 

PPARs and PGC-1α regulate mitochondrial aerobic metabolism, acting on different, though partially overlapping sets of genes. PPARs regulate expression of FAO genes, including *CD36/FAT*, *ACOX*, *SCAD*, while PGC-1α controls the expression of genes involved in oxidative phosphorylation. Treatment with BF of *Surf1^−/−^* mice increased expression of the PPAR isoforms present in the skeletal muscle, PPARα and PPARβ/δ. However, no increase in PGC-1α was observed in the skeletal muscles of BF-treated mice [98]. On the contrary, BF increased mitochondrial biogenesis and significantly increased expression of PGC-1α and battery of downstream its targets cytochrome c, TFAM, and subunits of ATP synthase in muscle, brown adipose tissue (BAT), and brain in mice with Huntington disease [99]. Treatment with BF rescued neuropathological features in the brain, increased motor activity, and muscle strength, prevented fiber–type switching in muscles, attenuated vacuolization in BAT and increased survival rate in Huntington mice. BF also was found to interact with hemoglobin and lower its affinity to oxygen. However, it is not clear whether the pharmacological doses of BF can achieve a concentration of the drug in erythrocytes sufficient to benefit the oxygen transport capacity [100,101].

Recent studies demonstrated the therapeutic effectiveness of BF to attenuate left-ventricular defects in the mouse model of Barth syndrome (BTHS). Barth syndrome is an X-linked rare genetic disease that is manifested by dilated cardiomyopathy, muscle weakness, and exercise intolerance. Causative gene is *TAZ* that encodes mitochondrial cardiolipin transacylase, tafazzin, and mutations in *TAZ* results in a deficiency of the essential mitochondrial phospholipid cardiolipin. Intake of BF with diet during the 4-month period in daily doses of 60–80 mg/kg (0.05% in rodent diet) or 600–800 mg/kg (0.5% in rodent diet) effectively prevented the development of systolic dysfunction and cardiomyopathy in *TAZ* knockdown mice [95,102]. Surprisingly, improvement of systolic function in mice treated with a high-dose (0.5%) of BF was accompanied by a simultaneous reduction of cardiolipin in the heart that can be explained by an increased number of mitochondria with a reduced content of cardiolipin [95].

Differential transcriptomic analysis of hearts demonstrated that treatment with a low dose (0.05%) of BF resulted in robust activation of genes involved in a wide-spectrum of biological processes that included metabolism of fatty acids, ketone bodies, amino acids and glucose, metabolism of proteins, mitochondrial protein transport, RNA metabolism, gene expression, DNA repair, chromatin organization, immune system, and organelle biogenesis and maintenance [102]. Bezafibrate failed to ameliorate the exercise intolerance phenotype in BTHS mice. However, when treatment with BF was combined with voluntary daily exercise on the running wheel, BF’s effect on exercise capacity in BTHS mice was significantly potentiated. The mechanisms underlying this synergistic effect of BF with everyday voluntary exercise are unclear. Apparently, exercise alters the epigenetic landscape in skeletal muscles and facilitates transcription of PPAR target genes thereby enhancing cellular metabolic plasticity. 

### 4.2. Clinical Studies

Currently, 41 clinical trials are registered to investigate the therapeutic efficiency of numerous PPAR agonists in various diseases worldwide (www.clinicaltrials.gov). Among those, eight trials have been completed, and no serious adverse effects have been reported. Among 41 trials, 13 are aimed to elucidate the efficacy of PPAR agonists in cardiac diseases. To date only one phase-2 clinical trial investigating the therapeutic potential of PPARγ agonist rosiglitazone in patients with congestive HF has been completed (NCT00064727), however, findings have not yet been reported. 

Recently, comprehensive systemic research of major cardiovascular disease prevention trials with fibrates was performed [103,104]. In these trials, data associated with the effects of clofibrate, which is no longer in use, were excluded from the analysis. Moderate-quality evidence from six primary prevention trials with 16,135 participants (8087 in the intervention group and 8048 in the placebo group) suggested that fibrate therapy reduced the combined outcome of death due to cardiovascular disease, heart attack, or stroke by 16% [103]. 

The Bezafibrate Infarction Prevention (BIP) study was initiated in 1998 with a total of 3090 enrolled participants (1548 in the intervention group, 1542 in the placebo group). The goal of this trial was to evaluate whether treatment with BF was effective in preventing MI injury and death in coronary artery disease patients. At 8.2 years of follow-up, there was an 18% risk reduction of major cardiac events (occurrence of cardiac death or nonfatal MI) (*p* = 0.02) [105]. Prolonged, 16 years of follow-up, showed that there was an 11% reduction (*p* = 0.06) in mortality in patients that were treated with BF [106,107,108]. BF had no therapeutic outcome on the risk of coronary heart disease and stroke in men with lower extremity arterial disease, although reduced the incidence of non-fatal coronary events in men aged 65 years or older [109]. 

Patients, who develop metabolic syndrome are at particularly increased risk of myocardial infarction (MI). The efficacy of BF to prevent MI was analyzed on the subgroup of patients from BIP study, who had developed metabolic syndrome (740 patients from BF group and 730 patients from the placebo group). The rate of nonfatal MI was significantly lower in patients in the BF group (9.5% and 13.8% in BF and placebo groups, respectively; *p* = 0.009). The decrease in MI incidence in patients taking BF was reflected in a trend to a 26% reduction of cardiac mortality rate (*p* = 0.056) [110]. 

Existing trials are mainly directed towards the studies of lipid-lowering and anti-atherosclerotic effects of PPAR agonists, whereas the bioenergetics actions of PPAR agonists on energy metabolism in cardiomyocytes remain less investigated. Although no clinical trial has prospectively studied the effects of PPAR agonists in patients with HF, there are several compelling evidences that PPAR agonists can improve clinical outcomes in HF. 

Treatment with BF in combination with ursodeoxycholic acid had a beneficial effect in patients with primary biliary cholangitis, a progressive liver disease, compared to a control group that was treated with ursodeoxycholic acid alone [111].

Studies including the cohort of six patients with the myopathic form of CPT-2 deficiency showed that six-month-long treatment with BF (200 mg three times a day) markedly upregulated CPT-2, increased oxidation rates of the long-chain fatty acids, decreased muscle pain and increased physical activity in all BF-treated patients [112]. BF failed to improve FAO in skeletal muscles and exercise tolerance in patients with CPT-2 and very long-chain acyl-CoA dehydrogenase deficiencies [92]. The authors ascribed the lack of effect FAO to the suppression of lipolysis by BF. However, an alternative explanation is that high plasma insulin in BF-treated patients had markedly inhibited lipolysis, hence hindering any increase of FAO and masking the effects of BF [113]. In vitro studies of patient cells with very long-chain acyl-CoA dehydrogenase and CPT-2 deficiencies revealed that treatment with BF is beneficial in cells of mildly affected patients that retain residual FAO capacities. In contrast, no increase in FAO capacities is expected in response to BF if the gene mutations impact the catalytic site or lead to highly unstable or severely misfolded proteins [96,114]. A phase 2 clinical trial of BF in BTHS patients (CARDIOMAN) is underway at University Hospital in Bristol, England.

## 5. Conclusions

PPARs play a central role in the pathogenesis of cardiac hypertrophy and HF and thereby, represent a potentially attractive therapeutic target for the treatment of these diseases. PPAR agonists, particularly PPARα and PPARβ/δ agonists appear to stimulate FAO and energy metabolism in cardiomyocytes. Subsequently, improved cellular energy homeostasis in the heart attenuates systolic dysfunction in HF patients as well as in animal models of cardiomyopathy and HF. Skeletal muscle appears to be more resistant to the treatment with PPARα and PPARβ/δ agonists. Full understanding of the therapeutic potential of PPAR agonists requires more detailed studies using various animal models of cardiac diseases. The effects of PPAR agonists on cellular transcriptional and epigenetic landscapes as well as activation/inhibition of individual PPAR isoforms on cellular metabolic and signaling systems need to be evaluated in detail using systems biology approaches. 

## Figures and Tables

**Table 1:** Bezafibrate trials using mouse genetic models with mitochondrial defects.

Disease Model	Tissue Studied	BF Dose	Effects	Ref.
OXPHOS defect: *Surf1^−/−^*	Muscle	0.5% (0.6–0.8 g/kg)	Weight loss, hepatomegaly. Increased expression of FAO genes, PPARα and PPARβ/δ.	[98]
OXPHOS defect: *Cox15^−/−^*	Muscle	0.5% (0.6–0.8 g/kg)	Toxic, mitochondrial myopathy, excessive apoptosis.	[98]
Huntington disease: *Htt*-ex1 (R6/2)	Brain, Muscle, BAT	0.5% (0.6–0.8 g/kg)	Attenuated neurodegeneration in brain, prevented muscle–type switching. Increased exercise capacity and muscle strength, increased vacuolization in BAT, and extend survival.	[99]
Premature aging: mtDNA polymerase γ^−/−^	Skin, Spleen	0.5% (0.6–0.8 g/kg)	Delayed hair loss and restored skin structure. Improved spleen size and structure.	[93]
BTHS: TAZ knockdown	Heart	0.5% (0.6–0.8 g/kg)	Preserved cardiac systolic function. Reduced cardiolipin level in mitochondria.	[95]
BTHS: TAZ knockdown	Heart, Muscle	0.05% (0.06–0.08 g/kg)	Restored cardiac systolic function. Ameliorated exercise intolerance phenotype when treatment was combined with everyday voluntary exercise.	[102]

## Abbreviations

AMPK	AMP kinase
BAT	brown adipose tissue
BF	bezafibrate
BTHS	Barth syndrome
CHD	coronary heart disease
CPT	carnitine palmitoyltransferase
CyP-D	cyclophilin D
FAO	fatty acid oxidation
HF	heart failure
MI NRF	myocardial infarction nuclear respiratory factors
PGC-1α	proliferator-activated receptor gamma coactivator-1 alpha
PPAR	peroxisome proliferator-activated receptor
TAC	transverse aortic constriction
